# Predicting Electrical Conductivity in Bi-Metal Composites

**DOI:** 10.3390/ma17205049

**Published:** 2024-10-16

**Authors:** Daniel N. Blaschke, John S. Carpenter, Abigail Hunter

**Affiliations:** Los Alamos National Laboratory, Los Alamos, NM 87545, USA; carpenter@lanl.gov (J.S.C.); ahunter@lanl.gov (A.H.)

**Keywords:** electrical conductivity, composite materials, crystallographic defects

## Abstract

Generating high magnetic fields requires materials with not only high electric conductivity but also good strength properties in order to withstand the necessarily strong Lorentz forces. A number of bi-metal composites, most notably Cu/Nb, are considered to be good candidates for this purpose. Here, we generalize our previous work on Cu/Nb in order to predict, from theory, the dependence of electric conductivity on the microstructure and volume fraction of the less conductive component for a number of other bi-metal composites. Together with information on strength properties (taken from previous literature), the conductivity information we provide in this work can help to identify new promising candidate materials (such as Cu/Nb, Cu/Ag, Cu/W, …) for magnet applications with the highest achievable field strengths.

## 1. Introduction

Studying fundamental questions and behaviors (such as phase transformations) in a wide range of materials systems such as semi- and super-conductors, quantum matter and thin films, often requires the creation of ultra-high magnetic fields (>80 T). The current world record for pulsed magnetic fields is 100 T, which was set about a decade ago [1]. While there is high demand for the use of such ultra-high magnetic field resources, access can still be limited for multiple reasons, one of them being the material properties of the conductive wire used within the magnet [2]. Currently, the 100 T pulsed field magnetic at the National High Magnetic Field Laboratory utilizes a Cu/Nb nano-composite wire for this purpose [2]. This wire is manufactured using an accumulative drawing and bonding (ADB) method that makes it difficult to produce consistent material properties from batch to batch and over the relatively long lengths required in the magnet [2]. Variations in material properties can cause experiments to either outright fail or have sub-par performance, resulting in costly delays and limitations in the availability of this resource.

These conductive wires are required to have a unique set of material properties. Particularly, in order to withstand the necessarily high Lorentz forces, the conductive winding wire utilized in these applications must have both high conductivity as well as exceptional strength properties [3,4,5,6,7]. For example, the current Cu/Nb wire has an ultimate tensile strength (UTS) of ∼1 GPa and an electrical conductivity of ∼70% IACS (International Annealed Copper Standard) at room temperature [8,9]. Furthermore, the wire needs to be fairly ductile as it is wound into coils to be used in the magnet [2]. Finally, these ultra-high pulsed field magnets operate at low temperatures (∼77–400 K [2]), so the material properties of the wires must be maintained through a range of temperatures. For example, at 77 K, Cu/Nb can exhibit very high electrical conductivities, ∼300% IACS and an UTS ranging from ∼1–1.4 GPa [8,9]. New composite materials with improved material properties regarding electrical conductivity and strength help to open the door for even higher pulsed magnetic fields, which would enable new experimental possibilities for several different classes of materials.

Promising material candidates for this purpose that so far have been identified include Cu/Nb [3,8,10,11], Cu/stainless steel [10,11], Cu/Cr [12], Cu/W [13,14], Cu/Ta [15], and Cu/Ag [4,16,17]. All of these examples are two-phase composites, which are fabricated using severe plastic deformation (SPD) methods such as ADB or accumulative roll bonding (ARB). Because it is currently used in ultra-high pulsed field magnets, the majority of previous literature has been focused on understanding and optimizing Cu/Nb. Thus, the material properties of Cu/Nb naturally become the baseline performance on which the community aims to improve. As mentioned, several other candidate materials have been investigated, and with the recent work on multi-principal elements and high-entropy alloys, one can imagine a vast design space in which to explore.

The focus of this current work is on bi-metal composites, and as such, we continue our previous study [18], of understanding and predicting electric conductivity based on the microstructure. Together with a separate study of material strength (not included here), this endeavor will help to identify new materials with even more optimized conductivity/strength properties for magnet applications. In particular, we generalize our previous theoretical study of Cu/Nb to a number of other bi-metals and identify the volume fraction the second (less conductive) component should not exceed in order to maintain good conductivity. A related recent numerical study of Cu/Nb composites can be found in Ref. [19].

The paper is organized as follows: We start in Section 2 by reviewing the modeling techniques, i.e., the phase field framework and the models predicting the contributions of the various microstructures to electric resistivity. We then proceed in Section 3 by presenting our simulation results for the electric conductivity of a number of bi-metals. Strength properties of those bi-metals are assembled from the literature, and we give an overview of promising candidates of strong and conductive bi-metals in Section 4.

## 2. The Model

Like in our previous work [18] and following [20], we consider local charge density $\rho_{e}$ as our order parameter within a phase field approach and (within our simulations) must apply an external electric field $E_{\mathrm{ex}}$. The local electric field E is generated by the spatial distribution of charge density $\rho_{e}$ and the externally applied electric field, i.e.,

(1) $$\begin{array}{cc} E(x) & =E^{\mathrm{ex}}-\frac{i}{\varepsilon_{0}}\int \frac{d^{3}k}{{\left(2\pi \right)}^{3}}\frac{{\tilde{\rho }}_{e}\left(k\right)}{k^{2}}ke^{ikx}\;, \end{array}$$

where ${\tilde{\rho }}_{e}$ is the Fourier transform of $\rho_{e}$. The local current density j is then given (via the microscopic version of Ohm’s law) by

(2) $$\begin{array}{cc} j_{i} & =\sigma_{ij}E_{j}\left(x\right)=\sigma_{ij}E_{j}^{\mathrm{ex}}-\frac{i\sigma_{ij}}{\varepsilon_{0}}\int \frac{d^{3}k}{{\left(2\pi \right)}^{3}}\frac{k_{j}}{k^{2}}{\tilde{\rho }}_{e}\left(k\right)e^{ikx}\;, \end{array}$$

where conductivity σ may take a tensorial form to account for anisotropy. For simplicity, however, we assume an isotropic approximation for $\sigma_{ij}\approx \sigma \delta_{ij}$.

The law of charge conservation, ${\dot{\rho }}_{e}=-\nabla j$, is related to the Ginzburg-Landau equations within a phase-field formulation. Hence, evolving this equation will give us a final charge density distribution, which via Equations (1) and (2) yields a current distribution j(x), i.e.,

(3) $$\begin{array}{cc} {\dot{\rho }}_{e} & =-\partial_{i}\left(\sigma E_{i}\left(x\right)\right)\\ \; & =-\partial_{i}\left(\sigma E_{i}^{\mathrm{ex}}\right)-\frac{\sigma }{\varepsilon_{0}}\int \frac{d^{3}k}{{\left(2\pi \right)}^{3}}{\tilde{\rho }}_{e}\left(k\right)e^{ikx}+\frac{i}{\varepsilon_{0}}\left(\partial_{i}\sigma \right)\int \frac{d^{3}k}{{\left(2\pi \right)}^{3}}\frac{k_{i}}{k^{2}}{\tilde{\rho }}_{e}\left(k\right)e^{ikx}\;. \end{array}$$

The experimentally measured macroscopic conductivity is then determined by spatially averaging j and the macroscopic version of Ohm’s law:

(4) $$\begin{array}{cc} \langle j_{i}\rangle & =\sigma^{\mathrm{eff}}E_{i}^{\mathrm{ex}}\;. \end{array}$$

In order to model local conductivity σ, we assume Matthiessen’s rule [21] holds, i.e., electric resistivity ρ=1/σ is the sum of bulk resistivity $\rho_{0}\left(T\right)$ and additional contributions to the bi-metal interfaces ($\rho_{i}$), grain boundaries ($\rho_{\mathrm{gb}}$), and average dislocation density $\rho_{d}$. We neglect sub-leading contributions from vacancies, interstitial atoms, and other types of defects, assuming they are not prevalent in our samples. The three contributions to ρ can be modeled as follows [18,22,23,24,25]:

The probability of scattering electrons at a bi-metal interface is parameterized by model parameter p∈[0,1] and its value depends on the “roughness” of the bi-metal interface with typical values being in the vicinity of 0.5 [23]. The contribution to resistivity in the two phases of the bi-metal on either side of the interface stemming from such scattering events can be estimated as in the following equation.

(5) $$\begin{array}{cc} \rho_{\mathrm{if}} & =\rho_{0}\left(T\right)\left[\frac{3}{8}\left(1-p\right)\lambda_{0}\left(T\right)\frac{1}{d_{0}}\right]\;, \end{array}$$

where $d_{0}$ denotes the layer thickness, and $\lambda_{0}$ is the electron mean free path. Both $d_{0}$ and $\lambda_{0}$ can be different on either side of the interface. Note that after careful consideration, we realized the additional factor $2V_{f}/\left(1-V_{f}\right)$ depending on the volume fraction $V_{f}$ of the second phase (which is 1 for $V_{f}=1/3$) present in [18] should not be included. Also, we re-calibrated the fraction of layers ≥100 nm containing 2 grains (30% instead of 25%) as well as the standard deviation of the Gaussian distribution (50 nm instead of 40 nm); see the details given below in Section 3. Note that $\rho_{\mathrm{if}}$ increases significantly as the layer thickness shrinks to values competing with the electron mean free path.

Likewise, grain boundary scattering becomes important when grain sizes are small so that they are comparable to $\lambda_{0}$. The according contribution to electric resistivity is estimated from the following expression:

(6) $$\begin{array}{cc} \rho_{\mathrm{gb}} & =\rho_{0}\left(T\right)\left[{\left(1-\frac{3}{2}\alpha +3\alpha^{2}-3\alpha^{3}\ln\left(1+1/\alpha \right)\right)}^{-1}-1\right]\;,\\ \alpha & =\frac{\lambda_{0}\left(T\right)}{d}\frac{R}{1-R}\;, \end{array}$$

where the grain boundary reflection coefficient R∈[0,1] depends on the size and shape of the single crystal grains. Grains in an ARB material with thin layers are typically long and flat [26], so that *d* (the grain “size”) is taken to mean the grain thickness perpendicular to the layer orientation.

Dislocation density, finally, is parameterized by $\rho_{d}=R_{d}N_{d}$, where the “scattering power” $R_{d}$ is typically of the order of $10^{-25}$Ω$m^{3}$ [27] so that dislocation densities ($N_{d}$) below $10^{16}$ $m^{-2}$ lead to negligible contributions to electric resistivity. Experiments with Cu/Nb have shown [18] that typical dislocation densities are below or close to $10^{15}$ $m^{-2}$. Here, we assume this value for $N_{d}$ in our simulations, even though its effect is in the sub-percent level and thus very small.

## 3. Conductivity Results

**Figure 1:**
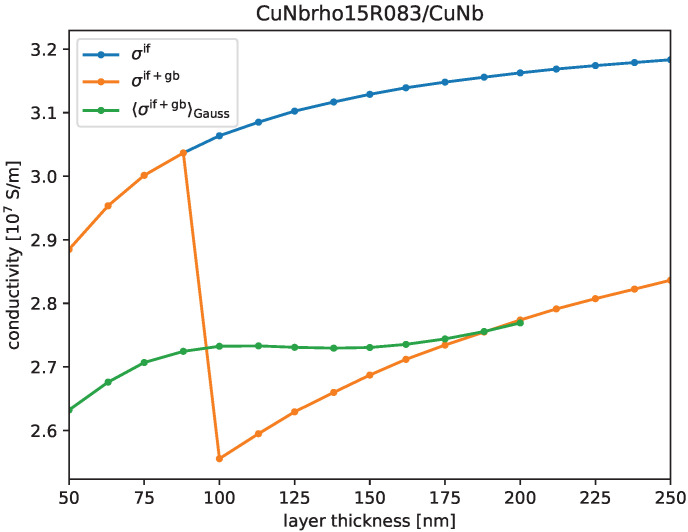
We show electric conductivity as a function of layer thickness for a Cu/Nb composite. The blue curve shows model results, only taking into account the effect of interface scattering. For the orange curve, we have assumed that 30% of the thicker layers (≥100 nm) have two grains across the layer thickness. The green curve shows the effect of having a distribution of layer thicknesses and was computed using a Gaussian distribution of the results shown in orange with a standard deviation of 50 nm. This rough calibration is based on our previous work, Ref. [18], using our own measured Cu/Nb data, which exhibited a range of layer thicknesses within each sample.

**Figure 4:**
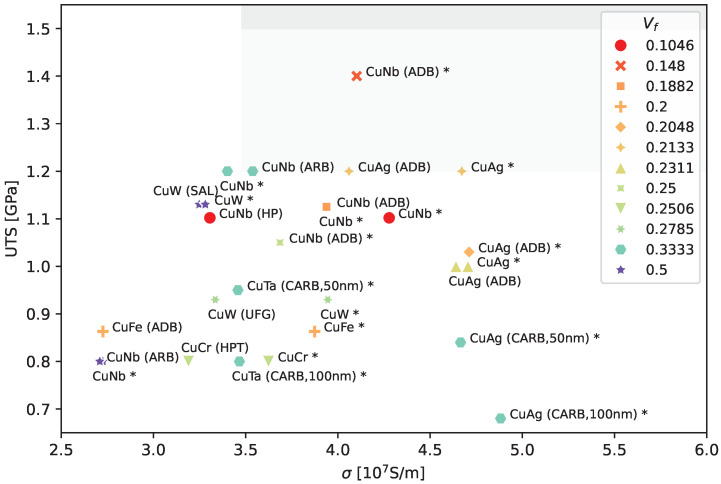
In this overview of strength and electric conductivity of various bi-metals, the shaded area indicates the requirements for generating magnetic fields of 100 T and beyond. The material and processing method are indicated in the marker annotations, where ADB refers to ‘accumulative drawing and bonding’, ARB denotes ‘accumulative roll bonding’, CARB refers to ‘cross accumulative roll bonding’, UFG denotes ‘ultrafine-grained’, HP refers to ‘hot pressed’, HPT is ‘high-pressure torsion’, and ‘SAL’ is ‘self-assembled lamellar architecture’. The volume fraction of the second metal is indicated in the legend. For some roll-bonded bi-metals, we also indicate the ‘nominal’ layer thickness, i.e., at a volume fraction of 1/3, the nominal layer thickness is that of the second metal and the Cu layer thickness is twice that number (or rather, two Cu layers are always bonded together). In cases where the electric conductivity was not reported, we use our own simulation result for the purpose of this plot and indicate so by a star ∗. Additionally, we compare our simulation results (for 100 nm Cu layers within ARB) to all cases where conductivity was reported in the literature (indicated by a star ∗ once more). Experimental data were taken from Refs. [10,22,32,33,36,38] (Cu/Nb), [10,39] (Cu/Fe), [15] (Cu/Ta) [40,41] (Cu/Cr), [7,16,34,42,43] (Cu/Ag) [14,35] (Cu/W).

In our previous work, we showed that a typical Cu/Nb bi-metal exhibits a distribution in layer thickness, rather than equally thick layers, an effect that is even more pronounced when the volume fraction $V_{f}$ differs from 0.5 [18,28]. Furthermore, for sufficiently thin layers, most of those layers only have one single crystal grain across the layer thickness, and a smaller fraction of layers of thicknesses beyond 100 nm exhibit two; less frequently, there are more grains across the thickness. Lacking details of the grain distribution, a rough first-order approximation, which led to good theoretical predictions for conductivity, was achieved by calibrating the fraction of layers thicker than 100 nm to have two grains across the thickness [29] and only one grain otherwise. Combining this assumption with a Gaussian distribution in layer thickness (which could be changed if new data became available) led to good agreement with measured conductivity values for Cu/Nb with average layer thicknesses up to 150 nm, as shown in our previous work [18]. Figure 1 illustrates these two steps: The blue curve within this figure shows the theory predictions when only one grain is present across each layer thickness (re-calibrated in anticipation of Figure 4 below). Adding two grains to 30% of thicker grains leads to a drop in conductivity in those thicker layers, as shown in orange. A Gaussian distribution over the layer thicknesses (calculated from the orange curve) yields the final prediction in green.

Assuming that the same ARB processing techniques lead to similar grain sizes, we now proceed to apply those same assumptions to predict conductivity for a number of other bi-metals of interest, in particular: Ag/Fe, Cu/Ag, Cu/Cr, Cu/Fe, Cu/Nb, Cu/Ta, and Cu/W. Note that more accurate predictions would require detailed knowledge of the grain distribution within each bi-metal, which would have to be measured. Lacking this knowledge, the next best thing we can do presently is to use the same calibration and assumptions that led to good agreement with our Cu/Nb experiments in Ref. [18].

**Figure 2:**
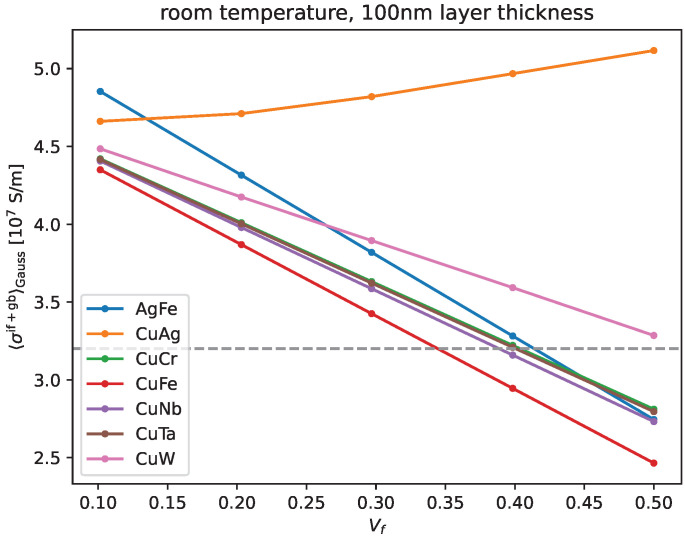
We show electric conductivity as a function of volume fraction for a number of bi-metal composites at room temperature. The mean value within a Gaussian distribution of layer thicknesses with 50 nm standard deviation for the first metal was 100 nm. Thirty percent of the thicker layers were assumed to have two single crystal grains across the thickness. The dashed gray line indicates the conductivity required in ultra-high magnetic field applications.

In Figure 2, we show our simulation results for those bi-metals at room temperature and with average layer thicknesses of the first metal of 100 nm as a function of volume fraction $V_{f}$ of the second metal (which, in most of our examples, is the less conductive metal). The second metal’s layer thickness equals that of the first metal only at $V_{f}=0.5$ and is typically thinner when the second metal’s volume fraction $V_{f}<0.5$ (see, e.g., [22,28]). Note that in cases where the second metal is significantly less conductive than the first, the volume fraction dependence is almost linear. The only exception here is Cu/Ag because both metals are good conductors in this case. Magnetic fields of about 100 T are achievable with bi-metals with an electric conductivity of 60% IACS together with an ultimate tensile strength (UTS) of 1.2 GPa [7]. In order to push the highest achievable magnetic fields well beyond 100 T (say up to 120 T), the UTS needs to be even higher [30,31], i.e., 1.5 GPa, without reducing electric conductivity below 55% IACS; this limit is indicated as a gray dashed line in Figure 2. For most of the listed bi-metals, this means a volume fraction not much higher than one-third should be considered.

**Figure 3:**
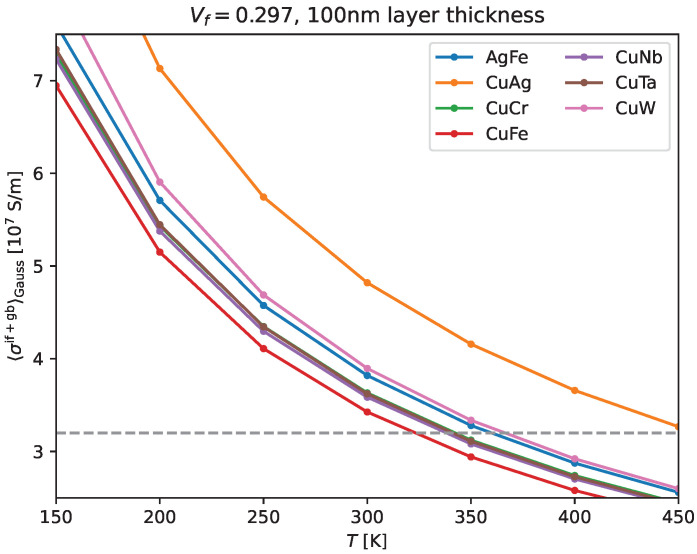
We show electric conductivity as a function of temperature for a number of bi-metal composites. The volume fraction of the second metal is $V_{f}\approx 0.3$ in all of these simulations. The average layer thickness was 100 nm, i.e., this was the mean value within a Gaussian distribution of layer thicknesses with 50 nm standard deviation. Thirty percent of the thicker layers were assumed to have two single crystal grains across the thickness. The dashed gray line indicates the conductivity required in ultra-high magnetic field applications.

We, therefore, focus on $V_{f}\approx 0.3$ for Figure 3, where we show the temperature dependence of electric conductivity for all bi-metals considered in this work. Again, 55% IACS is indicated in a dashed gray line, showing that this lower limit is exceeded even for the least conductive bi-metal, Cu/Fe, up to elevated temperatures of almost 350 K. We see that the temperature dependence in the range we simulated, i.e., 100–450 K (in 50 K increments), is very similar across all simulated bi-metals.

## 4. Review of Strength Properties

A number of bi-metals processed with various techniques have been studied with respect to their strength properties in the past. ADB Cu/Nb wires have been of particular interest with ultimate tensile strengths up to well beyond 1 GPa; see Refs. [10,32,33]. More recently, ARB as well as CARB (i.e., cross accumulative roll bonding, where every rolling step is undertaken perpendicular to the previous one) have also been studied for a number of bi-metals, including Cu/Nb, Cu/Ag, and Cu/Ta (among others). Strengths have been achieved up to 1.2 GPa [22]; see also Refs. [15,34]. Other processing techniques have been used as well, such as high-pressure torsion (HPT), e.g., Cu/Cr [12]. Apart from severe plastic deformation, researchers have also studied creating very strong bi-metals by reducing the grain sizes; for example, the authors of Ref. [35] achieved strengths above 0.9 GPa with ultrafine-grained Cu/W. Some of the studies mentioned above also measured electric conductivity of their bi-metal, such as Ref. [22], who achieved ∼61% IACS for ARB Cu/Nb, Ref. [12], who achieved 76 % IACS for HPT Cu/Cr, and Refs. [14,28,36]. Many others have focused solely on strength properties.

In Figure 4, we combine strength and conductivity data from the literature with our own conductivity simulation results. The material and processing methods of the experimental data from the literature are indicated in the marker annotations, where ADB refers to ‘accumulative drawing and bonding’, ARB denotes ‘accumulative roll bonding’, CARB refers to ‘cross accumulative roll bonding’, UFG denotes ‘ultrafine-grained’, HP refers to ‘hot pressed’, HPT is ‘high-pressure torsion’, and ‘SAL’ is ‘self-assembled lamellar architecture’. In all those cases where the electric conductivity was not reported, we use our own simulation result for the purpose of this plot and indicate so using a star ∗. Additionally, we compare our simulation results to all cases where conductivity was reported in the literature (indicated by a star ∗ once more).

Note that our simulations pertain to ARB materials, though we may expect those results to be close enough to the conductivity of ADB bi-metals, provided we simulated for layer thicknesses well above the electron mean free path for the (more conductive) matrix material. For those data where we have conductivity results from the literature, we compare them to our own predictions and see that (as expected) they are very close if the bi-metal was processed with ARB—most notably the conductivity reported by Ding et al. [22]. As for ADB processed materials, as strength is increased through additional drawing and bonding steps, conductivity decreases, and therefore, our ARB-optimized simulations overpredict conductivity for the ADB material (see Figure 4) since we do not account for the actual ADB microstructure in these cases. For this reason, the conductivity prediction for ADB Cu/Nb with UTS around 1.4 GPa shown in this figure is to be taken with a grain of salt: conductivity is likely somewhat lower than predicted.

For the purpose of Figure 4, we assumed 100 nm average layer thickness for the Cu phase in our simulations unless stated otherwise. Those results are meant to give readers an overview of bi-metals with promising strength and conductivity properties that are worth being studied further.

The main reason to study ARB instead of ADB is that ADB materials do not necessarily have the desired shape and have been unreliable despite their initially good properties, i.e., fractured filaments are introduced during the preparation process [22,36]. As we see from Figure 4, ARB materials have not achieved quite as high strength compared to ADB, but ARB has not been studied as long as ADB, and researchers are constantly improving the former [22,28,37].

From Figure 2, we see that a volume fraction of one-third or less for the less conductive material is necessary to achieve electric conductivities well above 55% IACS. Not surprisingly, many authors have thus focused on $V_{f}=1/3$, as shown in Figure 4. Clearly, ARB materials need to become even stronger to meet the 1.5 GPa UTS requirement for the next-generation magnets [30,31]. Our results and those from the literature indicate that Cu/Ag, CuW, and Cu/Nb exhibit conductivity and strength metrics that are closest to those needed for greater than 100 T magnets. However, a strength greater than 1.2 GPa (reported by Ding et al. [22] for Cu/Nb) still needs to be achieved while maintaining comparable electric conductivity (i.e., in order to move from the light-shaded into the dark-shaded area within Figure 4).

## 5. Conclusions and Outlook

Ultrastrong magnetic fields require materials with high electric conductivity as well as high UTS to withstand the necessarily strong Lorentz forces. Pushing the limits beyond 100 T has been a challenge, and in order to aid the identification of next-generation materials for these applications, we calculated electric conductivities from theory for a number of bi-metals processed by ARB. Furthermore, we presented a survey of previous strength and conductivity results from the literature. The latter conductivities were subsequently compared to our predictions (see Figure 4), which are fairly accurate for ARB, but (not surprisingly) often overpredict conductivity for ADB and otherwise processed bi-metals. Adapting our underlying model of microstructure together with accurate measurements of the actual microstructure will no doubt lead to more accurate predictions.

## Data Availability

The data presented in this study are available on request from the corresponding author. The data are not publicly available due to legal restrictions.
